# Comparison of additive and subtractive digital workflow for the accuracy of removable occlusal stabilization-splints – an in-vitro study

**DOI:** 10.1007/s00784-026-07211-0

**Published:** 2026-10-09

**Authors:** Anna-Lena Willmann, Joachim Schroeder, Tudor Radu Popescu, Karin Jepsen, Søren Jepsen, Pia-Merete Jervøe-Storm

**Affiliations:** 1 Department of Operative Dentistry and Periodontology, University Hospital Bonn, Welschnonnenstrasse 17, 53111 Bonn, Germany; 2 Private Practice, Auf der Schleide 75, 53225 Bonn, Germany

**Keywords:** 3D-printed, Milled, Periodontitis, Occlusal stabilization-splint, Tooth mobility, Digital

## Abstract

**Objectives:**

Patients with stage IV periodontitis often require additional rehabilitation of the compromised dentition due to anatomical and functional consequences of tooth loss. Removable occlusal splints can be helpful in these advanced cases. This in-vitro study compared the accuracy and internal fit of removable occlusal splints fabricated by 3D-printing and milling from a standardized STL-file.

**Materials and methods:**

A standard-tessellation-language (STL)-file was created from a scanned master-model of the upper jaw and used for production of forty identical splints. Twenty splints were 3D-printed and twenty were milled. Subsequently all splints were rescanned, new STL-files resulted, which were compared with the original STL-file. Accuracy of both methods were assessed at 42 predefined points. Distances from vestibular to vestibular (v-v) respectively palatinal to palatinal (p-p) between molars were compared. Mann-Whitney-test was used for comparison (*p* < 0.05).

**Results:**

Mean values were 43.9 ± 31.29 μm (3D) and 22.0 ± 18.46 μm (milled), with a significant difference between groups (*p* < 0.001, Mann-Whitney). The 3D technique resulted generally in a contraction (mean (SD) v-v: mean − 13.0 μm (± 92.16); p-p: -9.3 μm (± 123.03)). With the milled technique, a contraction vestibular-to-vestibular (-4.0 μm (± 53.63)), but palatinal-to-palatinal an expansion was measured (38.5 μm (± 33.86)). Vestibular, differences between groups were not significant (*p* = 0.862, Mann-Whitney), but palatinal at the second molar (*p* < 0.001, Mann-Whitney).

**Conclusion:**

Both techniques showed deviations comparable to previous in-vitro studies of digitally manufactured occlusal splints. Less deviations were found in posterior areas of the dental arch with milled manufacturing.

**Clinical relevance:**

Manufacturing of removable occlusal splints with CAD/CAM appears to be a precise and comfortable option.

## Introduction

Stage IV periodontitis is defined, among other things, by tooth drifting and flaring due to extensive attachment loss and tooth hypermobility due to secondary occlusal trauma. There is a high risk of tooth loss which leads loss of masticatory function [1–3].

Prognosis of periodontally compromised, hypermobile teeth with extensive CAL was improved through splinting, additionally patients’ comfort was reported to be enhanced [3–6]. A clinical trial on 100 patients in periodontal supportive therapy showed, that patients with parafunctional habits without a biteguard had a poorer tooth prognosis after 5 years, than those with a biteguard [7].

The effect of tooth stabilization should be reassessed after step 2 (non-surgical) and step 3 (surgical therapy). Sonnenschein et al. compared (2021) periodontal parameters and oral health-related quality of life (OHIP-G14) before and after step 2 therapy in two groups with or without splinting of anterior teeth. The authors reported that tooth splinting did not affect the relative bone loss, but in the splinted group OHIP-G14 scores three month after non-surgical therapy were improved [6]. However, there was a trend towards high plaque scores in the anterior area in both groups, suggesting that oral hygiene measures can be impaired due to a fixed splint. In another study splinted teeth showed long-term survival, despite the greater challenges to oral hygiene [8].

In case of tooth mobility and parafunctional habits, occlusal imbalance can be equalized by a removable occlusal splint, especially if occlusal adjustment would have to be performed in the early stages of periodontal therapy [9].

The manufacturing of such a splint is done either digitally (CAD/CAM) by means of an additive or a subtractive procedure; or conventionally with a thermoforming process or autopolymerized acrylics [10]. Resin systems are/were the primary material used for removable occlusal splints and can be manufactured in various ways – traditionally heat-cured polymethylmethacrylate (PMMA) or vacuum-formed thermoplastic resins [10, 11]. These processes can be more time-consuming as the CAD/CAM methods [10]. In the additive process, layers are added using light-controlled polymerization, with common techniques like stereolithography (SLA) and digital light processing (DLP) [12]. The subtractive method involves milling of a pre-polymerized PMMA block, which is supposed to have a more precise fit with lower dimensional deviations and do not require subsequent hardening. This eliminates the risk of polymerization shrinkage, which can positively impact the accuracy of the milled biteguards [13].

Another advantage is the digital scan, which, in contrast to a conventional dental impression, is very unlikely to have any negative effects on severely periodontally damaged teeth with high mobility. The digital scan is created as a STL file using a special software, afterwards send digitally to the manufacturing unit. Traditional impressions have been associated with numerous limitations which include complex workflow, partly lack of precise reproduction or the need to start over if an impression fails [14]. Besides the better control and improved accuracy of the directly obtained digital models, lost or broken appliances can easily be refabricated using the digital files from a database [15]. Furthermore, an in-vitro study showed that digital impressions achieved higher accuracy in mobile abutment teeth compared to conventional impressions [16].

Overall, a digital workflow may facilitate the management and reproduction of removable occlusal splints for periodontally compromised teeth.

Despite the increasing use of CAD/CAM technologies for the production of removable occlusal splints, previous studies have shown differences in accuracy depending on the manufacturing method, material, printing orientation, and measurement approach [17]. But the internal fit of the splints produced using the two techniques in the present study has not been sufficiently investigated in a standardized experimental context.

Therefore, the aim of this in-vitro study was to compare the dimensional accuracy and internal fit of removable occlusal splints manufactured by additive 3D printing and subtractive milling using a standardized master STL file. We hypothesized that the accuracy of fit and dimensional accuracy would not differ between the two manufacturing methods.

## Materials and methods

A maxillary Frasaco master-model (Frasaco, Tettnang, Germany) was scanned with the Zirkonzahn^®^ S900 Arti laboratory scanner (Zirkonzahn, Gais (BZ), Italy) to obtain a digital impression (scan). Based on this, an initial removable occlusal splint was designed with the Bite-splint application of the Straumann^®^ Cares^®^ Visual software (Straumann, Basel, Switzerland). This served as the original STL file (Standard Tessellation Language), as a base, from which forty identical splints were manufactured.

The design adhered to stabilization-splint requirements, maintaining a 2 mm thickness occlusal and 1 mm vestibular/palatinal. Production data was generated as an STL file, which describes the splint surface through triangulation [18].

### 3D printing – additive manufacturing

**Fig. 1:**
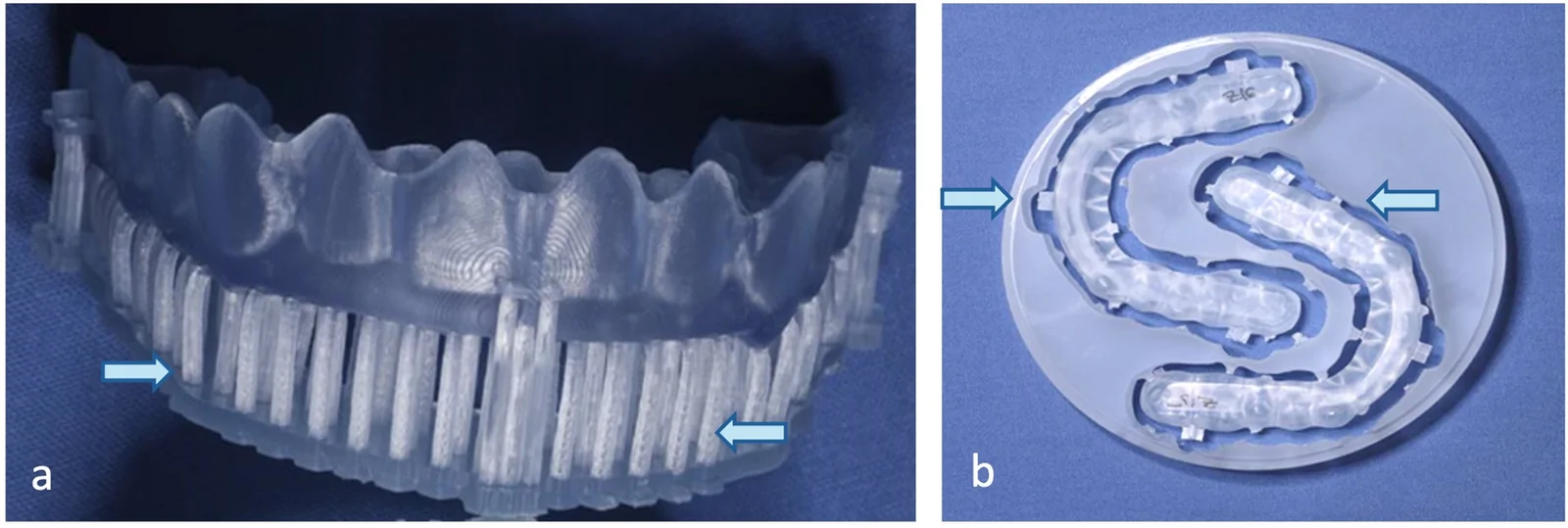
Comparison of manufacturing approaches: an additively manufactured splint (3D print) with support structures on the build platform (**a**, left) and two subtractively manufactured splints (milled) in the milling disk (**b**, right). Arrows show exemplary support structures

Twenty splints were designed using Straumann^®^ Cares^®^ Visual software (Straumann, Version: 13.1). The automatic-support-tool generated the design of the base and supports, positioning the splint at a 40° angle relative to the horizontal plane. The inside of the splint always pointed upwards, while supports connected the base-platform with the outer surface (Fig. 1a). The STL file was then forwarded to a P20 + printer (Straumann, Basel, Switzerland). For building of the splint KeySplint Hard (Keystone Industries GmbH, Singen, Germany), a material validated by Straumann for P series printers, was used. After printing, each splint was rinsed with 99% IPA (Isopropyl alcohol), washed in the P-Wash device (Straumann, Basel, Switzerland) with fresh IPA, inside facing down, and after 30 min of air drying subjected to post-curing for 15 min, using the P-Cure-System (Straumann).

The processing time was matched to the material through digital transmission of data. After the splints were cooled to room temperature, supports were removed. All steps were carried out according to manufacturer’s instructions.

### Milling – subtractive manufacturing

By the subtractive manufacturing process (milling) two splints could be produced simultaneously from a PMMA disc with a diameter of 95 mm (Fig. 1b) (Yamahachi PMMA Disk (Weithas GmbH, Lütjenburg, Germany)). For fabrication the Zirkonzahn^®^ M4 milling machine (Zirkonzahn, Gais BZ, Italy) was used. The milling machine features a 5 + 1 axis system with a 360° swiveling workpiece table, an optical tool recognizer for selecting appropriate milling head according to the geometry of the workpiece and is equipped with milling heads of varying diameters (1 mm, 2 mm, 3 mm). After separating the splints from the disk no further post-processing was required. Supports connected the splints with the discs lateral (Fig. 1b).

In both manufacturing processes, the support structures were located on the outside of the forty splints (Fig. 1a & b, arrows). Since all measurements were taken on the inner surfaces, the position of the support structures did not affect the results.

### Data collection

**Fig. 2:**
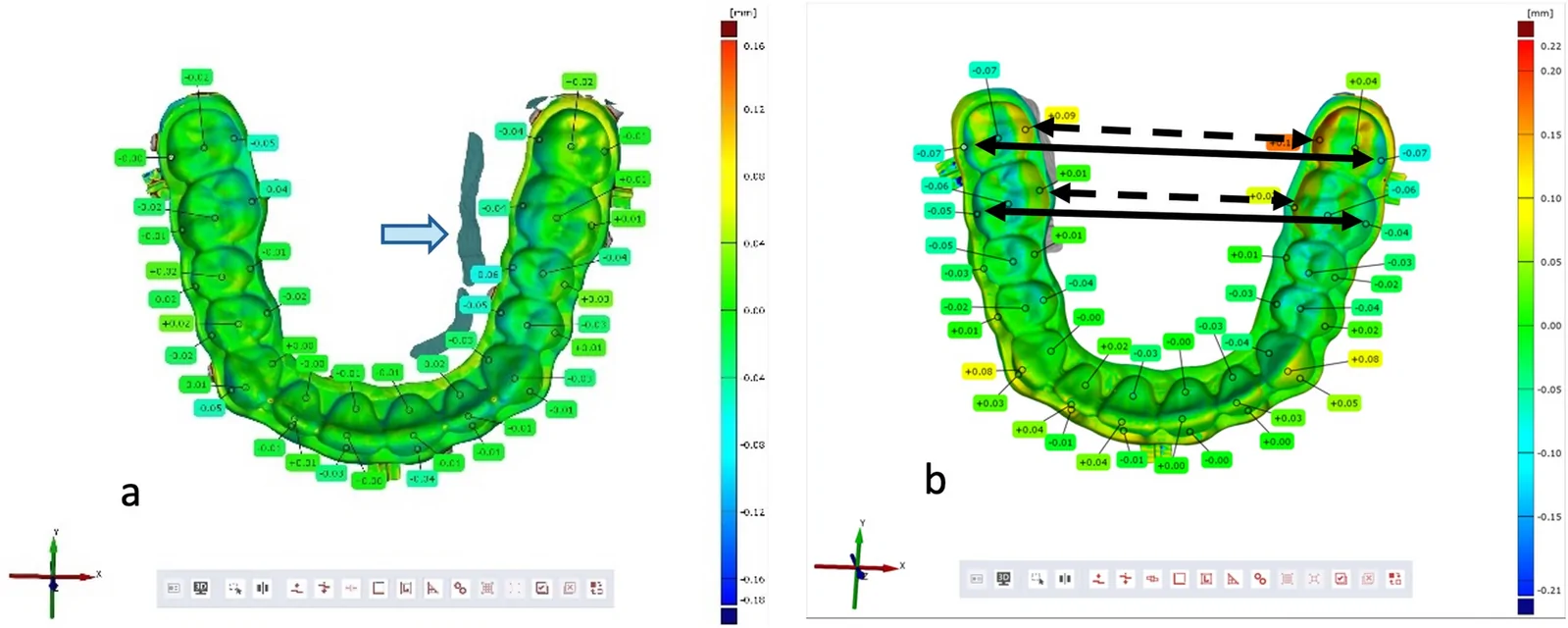
**a & b** Examples of the comparison of the 42 measurement points at a 3D-printed splint (**a**) and a milled manufactured splint (**b**) to original STL file in the GOM Inspect software (Zeiss, Jena, Germany). Deviations are recorded in millimeters and highlighted in color according to the legend on the right-hand side of each figure. Grey shadow in left picture (a) is an artefact (blue arrow). Black arrows in right picture (b) show vestibular measurement distances vestibular (straight line) and palatinal (stippled line) as measured at 1st and 2nd molars

After manufacturing, the 40 splints (20 additive, 20 subtractive) were scanned using Primescan AC (Dentsply Sirona, Charlotte, North Carolina). These new STL files were compared with the original STL file with Zeiss GOM Inspect software (Zeiss, Jena, Germany) using “Cloud-to-mesh” principle, in which the original STL file act as a point cloud and distances are measured as a perpendicular to the mesh of the new STL file. Measurements were taken virtually from the ‘inside’ of the splints at 42 defined points per splint (for each tooth three points: vestibular, palatinal, occlusal/incisal) on all 14 maxillary teeth, as the master-model had no third molars. With this method, the virtual fitting of the splint on the model could be determined. Deviations from the original STL file at each point were recorded in micrometers (Fig. 2a & b). For each point, the original STL file represents a zero point, implicating that the smaller the deviation, whether positive or negative, the more precise the splint. For clarification, a negative value represents a virtual gap, meaning not optimal fit. On the contrary, a positive value implicates the clinical necessity of adjustment of the splint, generally by removing excess material.

To assess the dimensional accuracy of the splints in the lateral direction, pairs of points were created. Each pair consisted of a left and a right point, both situated either vestibular or palatinal (Fig. 2b). Measurement points were identical with the vestibular respectively palatinal points selected for the first measurements. Occlusal/incisal measurement points were not considered. Distances between vestibular respectively palatinal measurements points at 1st and 2nd molars were compared to corresponding distances in the original STL file. A negative difference indicated a shortening of the measuring length as compared to the original file, while a positive value indicated a broadening.

### Statistical analysis

The primary objective of this study was to investigate whether the precision of the fit and dimensional accuracy of removable occlusal splints depended on the manufacturing method. For this purpose, STL files from 3D-printed and milled splints were compared with the original STL file.

Following null hypotheses were tested:

i. the precision of the fit does not depend on the manufacturing method (3D-printed or milled splints).
ii. there is no difference in dimensional accuracy between 3D-printed and milled splints.

In both manufacturing groups (3D-print or milled), mean, standard deviation (SD) and 95% confidence interval (CI) were calculated for each tooth in the respective three subgroups (vestibular, palatal, occlusal/incisal). Mann-Whitney test was used for comparisons (*p* < 0.05).

The absolute deviations were also analyzed descriptively using means, standard deviations and 95%CI. To assess the precision of the splints, distances in the original STL file between the vestibular (v-v) and palatal (p-p) points of the posterior teeth were utilized (17 to 27 and 16 to 26; Fig. 2b). The molars were chosen, because the greatest risk of dimensional deviations in the lateral was suspected to be posterior. Mann-Whitney test was used for comparisons (*p* < 0.05). Statistical software (SPSS 29.0 software package, SPSS Inc. Chicago, IL, USA) was used for the analysis.

A priori formal sample size calculation was not performed. The sample size was determined prior to the experiment in consultation with the ‘Institute for Medical Biometry, Informatics and Epidemiology; Medical Faculty of the University of Bonn’, taking into account the experimental design, planned statistical analysis, and sample sizes used in previous in vitro studies on the accuracy of CAD/CAM-fabricated occlusal splints. Based on these considerations, 20 samples were included per manufacturing group.

## Results

### Accuracy of measurement points

**Fig. 3:**
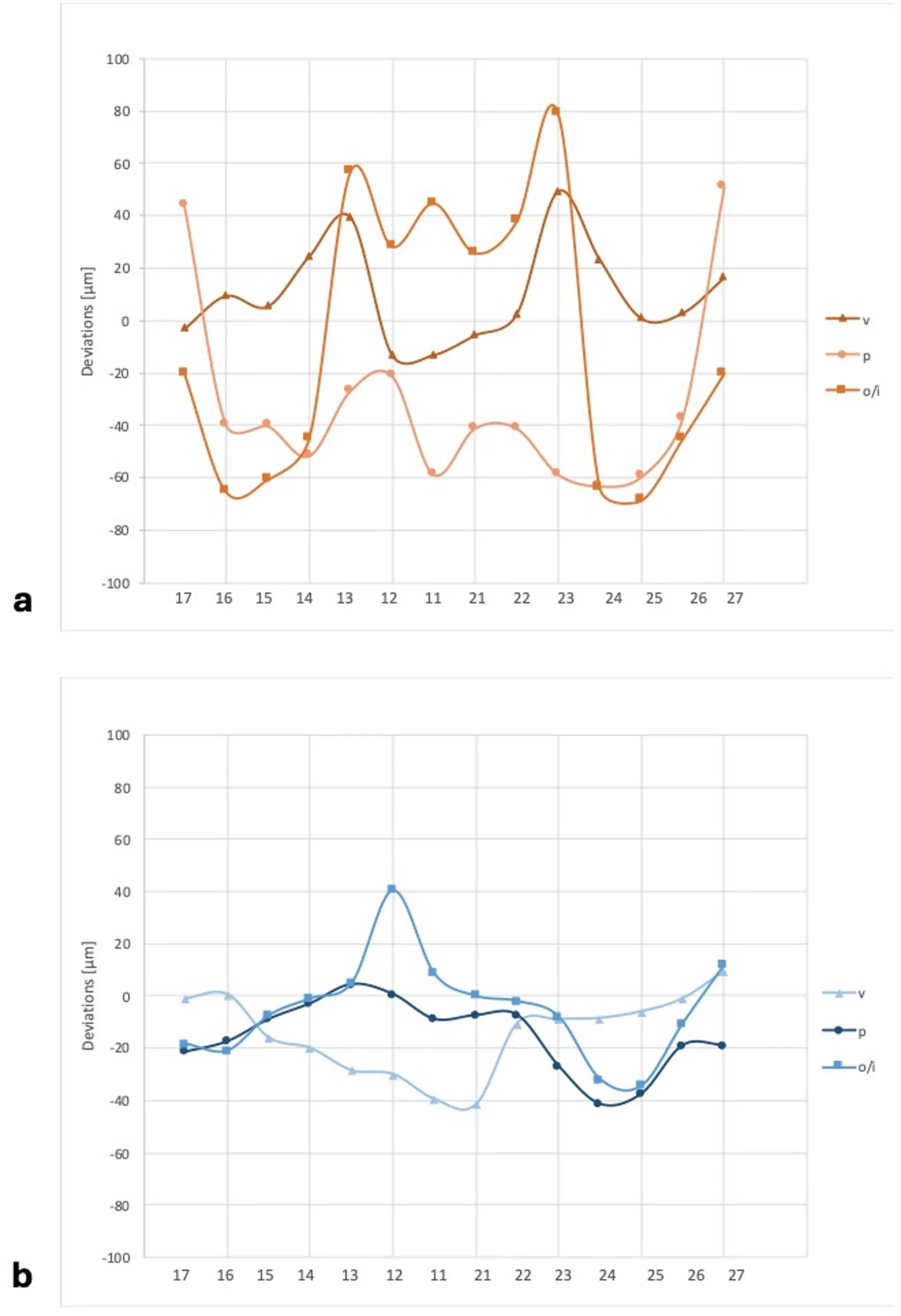
**a & b** Mean deviations (in µm) of both groups (3D-printed (Fig. 3a) or milled (Fig. 3b)), calculated for each tooth in the respective three subgroups (vestibular (▲), palatinal (●), occlusal/incisal (■)). X-axis (zero) represents the values of the original STL file

There were deviations from − 69 μm to + 79 μm when comparing *3D-printed splints* with the original (Fig. 3a). The y-axis represents the deviations from the original file in µm. A positive value indicates a too tight splint; a negative value indicates a too wide splint. Especially vestibular at the canines (13: +40 μm (±18.3); 23: +50 μm (±20.4)), palatinal at the second molars (17: +44 μm (±38.5); 27: +51 μm (±55.8)) and incisal in the canine-incisor area (range: +26 (±46.8) to + 79 μm (±56.6)) the printed splint was too tight (Fig. 3a). The largest single deviation appeared incisal of the left canine (23: +79 μm (±56.6)). Mean deviations of the vestibular, palatinal and occlusal/incisal results are shown in Fig. 3a, the zero line (x-axis) represents the values of the original STL file.

When comparing the *milled splints* with the original, less pronounced deviations were found (-42 to + 41 μm) (Fig. 3b). The largest positive deviation was located incisal at 12 (+ 41 μm (±21.6)). Negative differences were found at almost all measurement points, except for vestibular at 16 (+ 1 μm (±33.2)), palatinal at 12 (+ 1 μm (±8.6)) and 13 (+ 4 μm (±13.9)), and occlusal/incisal at 11 (+ 9 μm (±10.6)), 12 (+ 41 μm (±21.6)), 13 (+ 5 μm (±18.0)), and 27 (+ 12 μm (±24.0)). However, apart from incisal at the right lateral incisor, the deviations at these points were very small (< 12 μm).

A comparison of both manufacturing methods revealed significantly smaller deviations for each subgroup with the milled process in contrast to the results of the 3D-printed (*p* < 0.016, Mann-Whitney). When all measurements from the subgroups were combined, this was significant too (*p* < 0.001, Mann-Whitney). Herewith the null hypothesis i. was rejected.

**Fig. 4:**
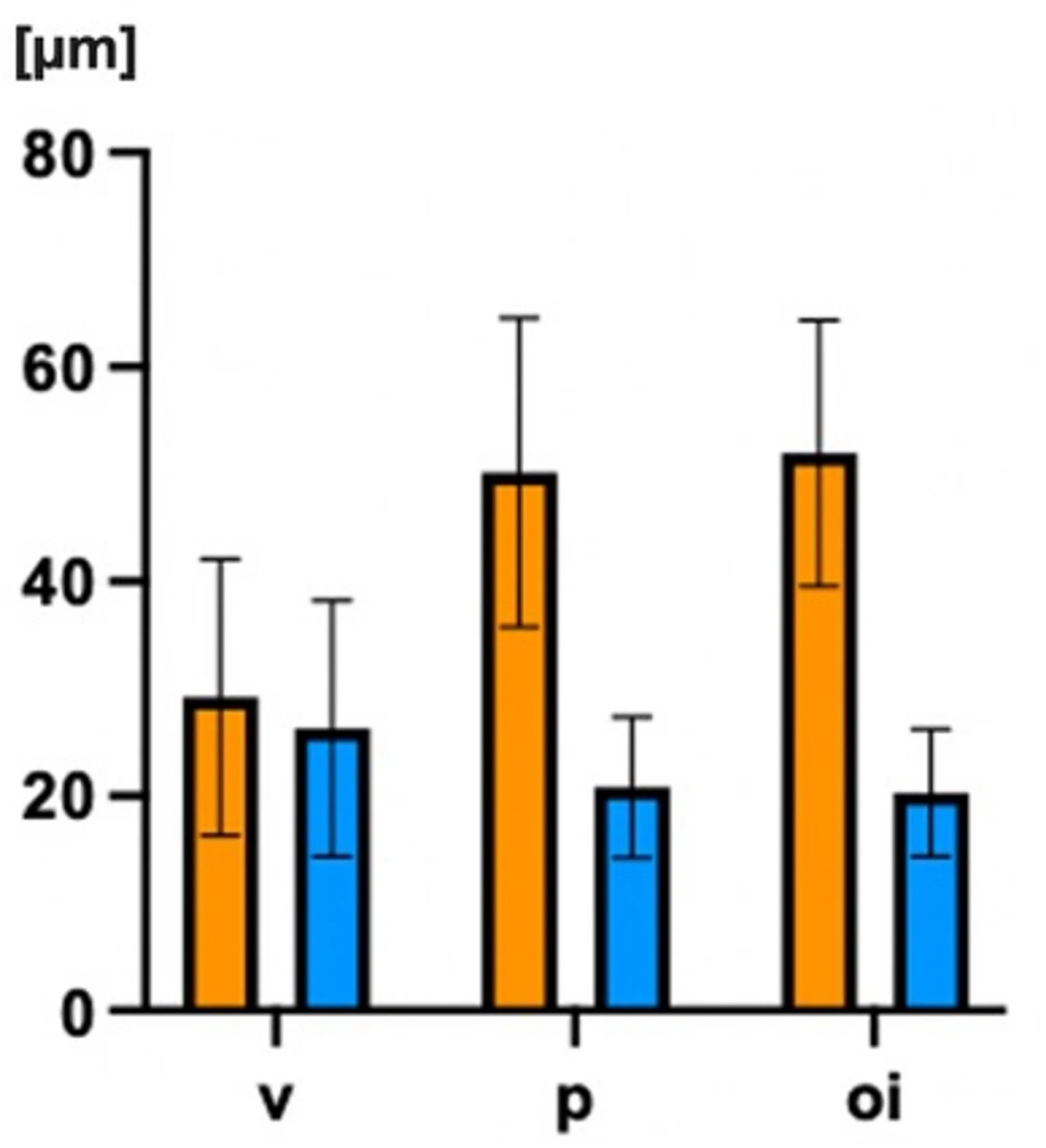
Average absolute values of vestibular, palatinal and occlusal/incisal deviations of 3D-printed splints (orange) and milled splints group (blue) recorded in micrometers

To be able to compare the dimensional accuracy of both manufacturing processes, regardless of whether the splint was too tight (positive value) or too wide (negative value), mean absolute values of the measurements of all twenty splints in each group for each tooth, divided into the three subgroups, were generated (Fig. 4). A comparison of the absolute values from the 3D-printed and the milled splints revealed larger deviations in the printed splint group, especially palatinal (3D-print 50.4 μm (±30.68); milled 19.2 μm (±16.22); *p* < 0.001, Mann-Whitney) and occlusal/incisal (3D-print 52.1 μm (±32.45); milled 20.3 μm (±16.46); *p* < 0.001, Mann-Whitney).

The greatest deviations of the milled splints were found on the vestibular surfaces, which were not significant but slightly smaller than those of the 3D-printed splints (milled 26.5 μm (± 21.44); 3D-print 29.1 μm (± 24.91); *p* = 0.403, Mann-Whitney) (Table 1).

**Table 1:** Mean deviations of absolute values (in µm) from the 3D-printed and the milled splints

Group	Additive process (3D print)			Subtractive process (milled)		
Subgroups	mean	SD	95% CI	mean	SD	95% CI
Vestibular	29.1	24.91	26.18–32.04	26.5^ns^	21.44	23.94–28.99
Palatinal	50.4	30.68	46.78-54.00	19.2*	16.22	17.31–21.12
Occlusal/incisal	52.1	32.45	48.25–55.89	20.3*	16.46	18.35–22.22
Overall	43.9	31.29	41.71–45.98	22.0*	18.46	20.74–23.24

*SD* standard deviation; 95%; *CI* 95% confidence interval; Overall: pooled results (vestibular, palatinal, occlusal/incisal). Comparison between groups: *p < 0.001; ^ns^not significant (*p* = 0.403, Mann-Whitney)

Overall (vestibular, palatinal and occlusal/incisal measurements combined), the mean deviation for 3D-printed splints was 43.9 μm (±31.29). The mean deviation found for the milled splints was 22.0 μm (±18.46), the difference between the results of 3D-printed and milled splints was significant (*p* < 0.001, Mann-Whitney). Herewith the null hypothesis i. was rejected.

### Deviations in Distances

For better understanding: Negative differences indicated a shortening of the measuring length, which can be understood as a contraction of the splint towards the center of the palate, while positive values indicated an extension away from the center of the palate (Fig. 2b; black arrows). Contractions were found vestibular at both molars and palatinal at the 2nd molar in the 3D-printed group (Table 2). Only palatinal at the 1st molar the distance from right to left measuring point was greater than on the original (76.5 μm (± 81.97)). In the group of milled splints, the distance between the vestibular measuring points at the second molar was smaller than originally measured (-8.5 μm (±58.33); Table 2). Otherwise, the distances between the measuring points on the milled splints were greater than the distances on the original.

**Table 2:** Descriptive statistics (mean, standard deviation, lower and upper confidence interval) for the deviations in distances of both groups between the vestibular respectively palatinal sides of the first and second molars

Site	Group	Additive process (3D print)			Subtractive process (milled)		
		mean	SD	95% CI	mean	SD	95% CI
vestibular	1st molar	-12.5	78.40	-49.19 to 24.19	0.5^ns^	49.58	-22.70 to 23.70
	2nd molar	-13.5	106.24	-63.22 to 36.22	-8.5^ns^	58.33	-35.80 to 18.80
	both	-13.0	92.16	-42.47 to 16.47	-4.0^ns^	53.63	-21.15 to 13.15
palatinal	1st molar	76,5	81.97	38,14 to 114,86	36.5^ns^	29.07	22.90 to 50.10
	2nd molar	-95.0	94.17	-139.07 to -50.93	40.5*	38.73	22.38 to 58.62
	both	-9.25	123.03	-48.60 to 30.10	38.50^ns^	33.86	27.67 to 49.33

*SD* standard deviation; 95%; CI 95% confidence interval. Negative results represent a “smaller” distance from right to left, positive values represent a wider splint right to left. Comparison between groups: *p < 0.001: ^ns^not significant (p *≥* 0.05, Mann-Whitney).

**Table 3:** Descriptive statistics (mean, standard deviation, lower and upper confidence interval) for the deviations in distances of both groups between the vestibular respectively palatinal sides of the first and second molars. Negative results represent a “smaller” distance (narrower splint) from right to left; positive values represent a wider splint right to left

Site	Group	Additive process (3D print)				Subtractive process (milled)		
		mean	SD	95% CI		mean	SD	95% CI
vestibular	1st molar	-12.5	78.40	-49.19 to 24.19		0.5^ns^	49.58	-22.70 to 23.70
	2nd molar	-13.5	106.24	-63.22 to 36.22		-8.5^ns^	58.33	-35.80 to 18.80
	both	-13.0	92.16	-42.47 to 16.47		-4.0^ns^	53.63	-21.15 to 13.15
palatinal	1st molar	76,5	81.97	38,14 to 114,86		36.5^ns^	29.07	22.90 to 50.10
	2nd molar	-95.0	94.17	-139.07 to -50.93		40.5*	38.73	22.38 to 58.62
	both	-9.25	123.03	-48.60 to 30.10		38.50^ns^	33.86	27.67 to 49.33

*SD *standard deviation; 95%; *CI *95% confidence interval. Negative results represent a “smaller” distance from right to left, positive values represent a wider splint right to left. Comparison between groups: *p < 0.001: ^ns^not significant (p *≥* 0.05, Mann-Whitney)

There were no significant differences between the two groups at the vestibular measurement points, neither at the first nor the second molar (*p* = 0.862; Mann-Whitney). At the palatinal measurement points, the difference between the groups was only significant for the second molar (first molar: *p* = 0.05; second molar: *p* < 0.001; Mann-Whitney). Herewith the null hypothesis ii. was only rejected for palatinal measurements at the second molar, but for all other distances the null hypothesis ii. was confirmed.

## Discussion

It was shown that with both techniques sufficient clinical precision can be achieved. Comparison of the STL files of the 3D-printed splints with the original STL file revealed deviations from − 69 μm to + 79 μm. Less pronounced deviations were observed when comparing the STL files of the milled removable occlusal splints with the original STL file (-42 to + 41 μm). In the absolute values the average precision of the milled splints was better than that of the 3D printed splints (milled: 22.0 μm (± 18.46); 3D printed: 43.9 μm (± 31.29)). The differences between both groups were significant (*p* < 0.001, Mann-Whitney), resulting in rejection of the first null hypothesis (i). The lateral distance from 1st to 2nd quadrant, was measured at the vestibular and palatinal aspects of the 1st and 2nd molars. Notably, there was no differences between the manufacturing methods vestibular at the molars (*p* = 0.862; Mann-Whitney). Only palatinal at the 2nd molar, there were significant differences between both methods (*p* < 0.001; Mann-Whitney).

The manufacturing information was provided in the commonly used STL file format, in which the surface is described by triangular facets. The accuracy depends on the distance between the points of the digital surface mesh of the splint [18]. The orientation of splints during printing and milling significantly influences accuracy too [17, 19]. Horizontal positioning on the printers buildplatform reduces the number of layers and cumulative deviations. However, over-curing effects may increase polymerization contraction due to light scattering [19]. Printing at a 40° angle, within the manufacturer’s recommended range, provides a balance between mechanical properties and surface quality [17]. Digital light processing (DLP) printers utilize a 385 nm light source to polymerize entire resin layers with high precision [20]. The positioning of splints within the PMMA blank can also affect accuracy in subtractive manufacturing. Preliminary trials of our group demonstrated that a large unsupported central area between splints, not connected to the outer frame of the blank, may lead to bending of the PMMA disk under its own weight. Therefore, a balanced positioning within the blank was selected for this study. This issue may explain recurring deviations between the first and second quadrants (Fig. 1). During milling, accuracy is primarily influenced by the milling tools. The precise reproduction of fine incisal edges is limited by the radius of the milling burs [17].

Post-processing of printed splints requires a technically sensitive workflow including washing, drying, and post-curing, as improper handling can significantly affect accuracy. The manufacturer’s instructions were strictly followed. Previous studies have demonstrated that correct post-processing of additively manufactured objects has a significant impact on both dimensional accuracy and mechanical properties [21, 22]. Our preliminary investigations indicated that storing splints with the internal surface facing downward during washing combined with sufficient drying time, significantly minimized residual monomer content, particularly in areas such as incisal edges and cusp tips. Therefore, special attention was given to proper post-processing in this study. Milled splints do not require post-processing; polymerization shrinkage is avoided by the use of pre-polymerized PMMA disks [13].

The Primescan enables high accuracy scanning of complete dental arches [23] and generates three-dimensional datasets based on triangulated surface points, translated to a new STL file. Particularly for capturing deep structures, such as the internal surface of splints, as its precision is minimally affected by variations in scanning depth [24]. Nevertheless, every impression — whether digital or conventional — contains a certain degree of error that may slightly influence measurements [25]. To assess potential chairside adjustments, specific measurement points were defined. The fabrication of full-arch dental appliances represents a considerable challenge [24, 26]. To account for possible polymerization shrinkage in the additive process, distances in the molar region were additionally measured.

A limitation of this study is that no formal a priori sample size calculation was performed. Prior to the experiment, 20 samples per manufacturing group were selected taking into account the standardized in vitro study design, the planned statistical analysis, and the sample sizes reported in previous studies on the accuracy of CAD/CAM-fabricated occlusal splints. Although this approach was considered appropriate for the present experimental comparison, the lack of a predefined effect size and formal power calculation means that insufficient performance to detect minor differences cannot be ruled out.

Previous studies have shown differences in the accuracy of additively and subtractively manufactured occlusal splints [17]. However, no universally established threshold for clinically acceptable internal adaptation currently exists. Therefore, the deviations observed in the present study should be interpreted as differences in dimensional accuracy rather than definitive differences in clinical fit.

Digital dentistry is gaining increasing importance. Intraoral scanning enables more efficient workflows and compact digital data storage [27]. Furthermore, errors in digital impressions can be identified immediately and corrected through localized rescanning, without the need for a complete repetition of the impression. Both additive and subtractive manufacturing processes provide straightforward and time-efficient workflows with consistent results, allowing easy reproduction of devices if needed [28]. However, current evidence comparing the accuracy of additive and subtractive manufacturing remains limited. Reymus et al. reported that milled splints exhibit greater accuracy than printed ones, mainly due to material properties [17].

It can be assumed that dimensional changes observed in additively manufactured splints are primarily due to polymerization shrinkage, as these splints exhibit contraction toward the center across the entire dental arch [29]. Other studies attribute dimensional changes in additively manufactured objects to increased water absorption compared to milled materials [30]. Water penetration between polymerization layers may induce movement within polymer chains, negatively affecting precision [22]. Another manufacturing-related difference between splints is surface roughness [31]. Printed splints exhibited higher surface roughness compared to milled splints [32]. The layer-by-layer fabrication process resulted in a characteristic stair-step surface pattern, particularly pronounced in areas with low inclination [18]. This not only increased surface roughness, with possible higher biofilm accumulation, but also reduced accuracy, as the surface was rather an approximation of the original design. Both issues can be minimized by reducing layer thickness, while this increases printing time [18, 26]. In addition to differences in accuracy, variations in mechanical properties depending on the manufacturing method have been reported [22]. A disadvantage of subtractive manufacturing compared to additive methods is the higher material consumption, as applications are milled from pre-polymerized blanks, resulting in substantial loss of unused material up to 70% [13, 33]. However, recent research has explored recycling methods for PMMA milling residuals to reprocess these materials for new dentures [34].

## Clinical relevance

To determine the most suitable manufacturing method for occlusal removable splints in clinical practice, factors beyond accuracy must be considered, including mechanical properties, hygiene capability, and clinical workflow. Digital workflows offer several advantages, particularly in the treatment of patients with periodontitis [35]. Periodontally compromised dentitions, especially as seen in patients with stage IV periodontitis, are characterized by tooth mobility and elongated teeth with numerous undercuts [1, 2, 36]. Tooth mobility resulting from bone loss and occlusal trauma often necessitates splinting and/or occlusal adjustment to reduce the risk of displacement functional loading [5, 37, 38]. In such cases, conventional impressions can be challenging and may lead to tooth displacement or even tooth loss [39]. In contrast, digital impressions allow pressure-free acquisition of the clinical situation, thereby reducing the risk of iatrogenic tooth movement during splinting procedures, which is a common issue in periodontal stabilization of mobile teeth [30, 39–41]. Accurate splints are essential to avoid unfavorable and unintended new positioning of the teeth within the splint and ensure patient comfort [5]. Particularly in patients with tooth mobility and parafunctional habits, occlusal splints can improve the prognosis of teeth [7, 42].

To directly evaluate efficiency and patient comfort, in-vivo studies involving patients with stage IV periodontitis are required. The increasing preference among both patients and clinicians suggests a growing shift towards digital workflow in clinical dentistry [43]. This technology may also be an advantage in managing periodontally compromised dentitions [40].

## Conclusion

Current evidence comparing the accuracy of additive and subtractive manufacturing remains limited. The results of the present in-vitro study do not allow any statements as to the clinical relevance of these differences. Both manufacturing methods offer efficient and reproducible workflows, allowing easy reproduction of splints in cases of loss or failure [28]. This could be confirmed in the present study.

Further clinical studies are required to investigate whether the observed deviations affect splint fit, patient comfort, tooth mobility, occlusal stability, or periodontal outcomes in patients with periodontally compromised dentitions.

## Data Availability

No datasets were generated or analysed during the current study.
